# Prevalence of online food delivery platforms, meal kit delivery, and online grocery use in five countries: an analysis of survey data from the 2022 International Food Policy Study

**DOI:** 10.1038/s41366-025-01771-z

**Published:** 2025-04-13

**Authors:** Rebecca Bennett, Clara Gomez-Donoso, Christina Zorbas, Gary Sacks, Christine M. White, David Hammond, Adyya Gupta, Adrian James Cameron, Lana Vanderlee, Alejandra Contreras-Manzano, Kathryn Backholer

**Affiliations:** 1 Global Centre for Preventive Health and Nutrition, Institute of Health Transformation, Deakin University, Geelong, VIC Australia; 2 School of Public Health Sciences, Faculty of Health, University of Waterloo, Waterloo, ON N2L 3G1 Canada; 3 School of Nutrition, Centre NUTRISS (Nutrition, Santé et Société), Université Laval, Quebec, QC G1V 0A6 Canada; 4 Center for Nutrition and Health Research, National Institute of Public Health, Cuernavaca, Mexico; 5 National Council for Humanities, Science and Technology, Mexico City, Mexico

**Keywords:** Nutrition, Public health

## Abstract

**Background:**

Online food retail use is rapidly increasing in popularity, and offers user-friendly apps, and new food delivery models, including online food delivery platforms, online grocery retailers, and online meal kit delivery. We aimed to: (1) quantify the prevalence of online food retail platform use by adults across Australia, Canada, Mexico, the United Kingdom and the United States, and to (2) assess the associations between sociodemographic and behavioural factors and use of online food retail platforms.

**Methods:**

A cross-sectional online survey was conducted with adults as part of the 2022 International Food Policy Survey (*n* = 19,877). We described the frequency of use and number of meals ordered using different online food retail and delivery platforms. Logistic regression models were fitted to assess associations between the use of online food retail and delivery platforms, and sociodemographic and behavioural factors (including age, sex, household composition, BMI, income adequacy, ethnicity, cooking skills, nutrition knowledge, and frequency of food preparation).

**Results:**

Online ordering was more prevalent in Mexico (72%), and in the United States (62%) in comparison with Australia, Canada, or the United Kingdom (45–56%). Overall, across all countries, 58% of participants used online retail and delivery platforms, most commonly online orders from restaurants (36% of participants), online supermarkets (28%), online meal kits (14%), online only grocery stores (11%), and online convenience stores (11%). The odds of using online restaurants was significantly higher for men (OR: 1.23, 95% CI: 1.14–1.33) and participants aged 18–29 (compared to those 60 years or older) (OR: 6.10, 95% CI: 5.34–7.00). Participants aged 18–29 also had the highest odds of using online convenience stores (OR: 7.51, 95% CI: 5.71–9.88). Participants living with primary school aged children had higher odds of using online supermarkets compared to those without children (OR: 2.56, 95% CI: 2.22–2.94).

**Conclusions:**

A substantial proportion of people are buying food online. Efforts to improve population diets need to ensure that online food retail platforms support good health and nutrition.

## Background

Frequent consumption of foods and beverages high in added sugars, saturated fats and sodium is a leading risk factor for weight gain and the development of diet-related non-communicable diseases such as type 2 diabetes, cardiovascular disease, and some cancers [1]. Globally, the prevalence of overweight and obesity has risen dramatically since the 1970s, particularly in high income countries such as Australia, the United Kingdom (UK), the United States of America (USA), and Canada [2]. Nations undergoing a transition towards diets that are increasingly dominated by unhealthy foods and beverages, such as Mexico, also experience a similar high prevalence of overweight and obesity, contributing to growing health concerns [3].

Online food retail, including websites and smart phone applications (apps), provide customers with easy access to food and beverages for either delivery or pick-up. Online food retail has been conceptualised into three types: online grocery retailers, online meal kit retailers, and online food delivery (OFD) platforms [4]. Online grocery retail allows customers to order groceries from supermarkets for either “click and collect” or delivery [5]. This category includes supermarkets that have physical stores, as well as “online only” grocery stores, such as Amazon and smaller independent stores catering to specific dietary needs. Online meal kit retailers offer a subscription service for delivery of pre-measured quantities of ingredients along with recipes for meal preparation [6]. OFD platforms such as Uber Eats, DoorDash, Just Eat, and SkipTheDishes, allow customers to order ready-to-eat meals from places like restaurants, cafes, and convenience stores [7]. Online food retail platforms are designed to be user friendly, featuring intuitive interfaces to facilitate transactions, and are becoming increasingly popular [8]. Globally, sales from online grocery have increased from US $110 billion in 2017, to US $390 billion in 2023 [9]. Meal kit subscription services made US $12.47 billion in revenue worldwide in 2022, with this predicted to increase to US $18.47 billion by 2027 [10]. In 2023 the global revenue of OFD platforms was US $390 billion [9]. The number of users of online food retail is also increasing rapidly, with OFD platform users rising from 806 million in 2018 to 1.9 billion in 2023 [11], and the combined number of users of online grocery and meal kit subscription services rising from 547 million in 2018 to 1.3 billion in 2023 [11].

Given their widespread use, online food retail is likely to have significant implications for diets and health [12]. OFD platforms have been criticised for promoting unhealthy food more frequently than healthy options, with most of the food outlets that sell through them considered unhealthy [13, 14]. Other studies examining online grocery retail have reported potential dietary health risks from increasing shifts away from purchases at physical stores, such as a reluctance to order fresh or perishable products (such as meat, and fruit and vegetables) online [15, 16]. However, online grocery retail may offer some health benefits related to increased access to healthy food for people with limited transport [17], and reduced purchases of unhealthy food online [18]. Online meal kit subscriptions have been identified as potentially health promoting because they may increase cooking knowledge and vegetable consumption, although equity concerns have been raised due to the relatively high cost of meals offered [6].

Despite the popularity of online food retail, and their potential implications for population health, to our knowledge, there is yet to be a comprehensive analysis of how the different online platforms are used, and how this differs across sociodemographic and behavioural characteristics. In this study, we aimed to:

1. Quantify the prevalence of online food retail platform use (OFD platforms, online grocery retailers, and online meal kit retailers) across adults in Australia, Canada, Mexico, the United Kingdom, and the United States.
2. Examine how online food retail use varies according to sociodemographic and behavioural factors.

## Methods

### Study design and data collection

We used cross sectional data collected in 2022 as part of the annual International Food Policy Survey (IFPS), in Australia, Canada, Mexico, the UK, and USA. The IFPS data collection methods have been previously described [19]. After being screened for eligibility, and providing consent, adults aged 18 years and older completed an online survey. Participants were recruited through the Nielsen Consumer Insights Global Panel, and their partner panels, in selected countries. Participants could complete the survey on their choice of desktop or laptop computers, or mobile devices including smartphones or tablets [19]. The median survey completion time across all countries was 36 min. Participants were compensated for their time with point based or monetary rewards.

The IFPS was approved by the University of Waterloo Research Ethics Committee (approval ORE #30829). All procedures were conducted in accordance with the ethical standards of the committee and the principles of the Declaration of Helsinki. Written informed consent was obtained from all participants prior to their participation in the study.

### Measures

#### Online food delivery platform use

Use of OFD platforms was assessed through questions that queried if participants had ordered meals/food/drinks from a restaurant or takeaway store, or snacks/food/drinks from a convenience store, online or using an app in the previous 30 days. Convenience stores refer to small retail stores that generally offer a more limited range of products, such as snacks, beverages, and basic groceries like milk and bread. Participants who responded affirmatively to these questions were then asked how often they had ordered from a restaurant or takeaway, and/or convenience store online or using an app in the previous 30 days, respectively, with the response options including “Less than once a week”, “Once a week”, “A few times a week”, “Every day”, “Don’t know”, and “Refuse to answer”. Participants who answered “Less than once a week”, “Once a week”, “A few times a week”, or “Every day” were considered users of online restaurants, or online convenience stores, respectively. Non-users were classified as those who responded that they had not ordered meals, food, or drinks from a restaurant or take-away online or using an app in the previous 30 days.

#### Online grocery retail use

To assess use of online grocery retail, participants were asked whether they had ordered groceries online or using an app from a supermarket or from an online-only grocery store (e.g. Amazon) in the previous 30 days. Participants who responded affirmatively were then asked how often they ordered in the past 30 days. Those who responded “Less than once a week”, “Once a week”, “A few times a week” or “Every day” were considered online grocery customers. Non-users were classified as those who responded that they had not ordered from an online supermarket or online-only grocery store, in the past 30 days, respectively.

#### Online meal kit delivery service use

Participants were asked if any of their food prepared at home included food purchased in the past 7 days from a meal kit delivery service (e.g. Hello Fresh or a country relevant brand) (yes/no), and were classified accordingly as users and non-users.

#### Sociodemographic, behavioural, and body weight variables

We assessed participant sex at birth, age, presence of children in the home aged less than 18 years, perceived income adequacy, cooking skills, nutrition knowledge, frequency of food preparation at home, ethnicity, and self-reported body mass index (BMI) as independent variables.

Age of participants was classified according to generation, with those aged over 60 years considered “Silent Generation and Baby Boomers”, those aged 45–59 “Generation X”, those aged 30–44 “Millennials”, and those aged 18–29 “Generation Z” [20]. Children in the home were classified according to the age of the youngest child. Participants whose youngest child was aged 4 years or younger were considered to have “preschool” aged children at home, those with youngest children aged 5 to 12 years were considered to have “primary school” aged children, and those with youngest children aged 13 to 18 years were considered “secondary school” aged.

Perceived income adequacy was assessed based on responses to a question asking how difficult or easy it was for their total monthly income to allow them to make ends meet. Those who reported that it was “difficult” or “very difficult” were considered to have “inadequate income”. Those who responded “very easy”, “easy” or “neither easy nor difficult” were considered to have “adequate income”.

Participants were asked to report their perception of their own nutrition knowledge, with this variable dichotomised as knowledgeable (those reporting “extremely knowledgeable”, “very knowledgeable” or “somewhat knowledgeable”) or not knowledgeable (“a little knowledgeable” or “not at all knowledgeable”). Similarly, participant’s self-perceived cooking skills were defined as “skilled” (those reporting “excellent”, “very good” or “good”) or “unskilled” (“fair” or “poor”). Participants were asked, “How often do you prepare a main meal for yourself or others?”. Participants who responded more than 3–4 times per week were classified as “frequent” with those who prepared food at home two or less times per week classified as “not frequent”. Ethnicity was self-reported using national country-specific measures, with categorisation as ethnic minority or majority for comparison across countries. BMI (kg/m^2^) was calculated from self-reported weight and height, and participant responses were classified as “obesity (BMI > 30 kg/m^2^)”, “overweight (BMI 25.5–29 kg/m^2^)” and “underweight/normal BMI (BMI < 25 kg/m^2^)” and included a “missing” category for survey participants who did not disclose their height and/or weight [21].

#### Study sample

A total of 35,214 participants completed the IFPS online survey. Respondents were excluded for the following reasons: ineligible region, invalid response to data quality question; below minimum survey completion time based on median survey time; and/or invalid responses to at least three of 20 open-ended measures (*n* = 8941). Among the remaining 26,273 participants, *n* = 5289 were part of an oversample of participants with lower educational attainment from Mexico and Mexican Americans from the United States and were not part of our analysis as they were not asked about online food purchasing [19]. Additional participants were excluded from the sample because their responses included inappropriate language (*n* = 5), or they responded “don’t know” or “refuse to answer” to key variables of interest including those relating to use of online food retail platforms (*n* = 284) and sociodemographic or behavioural questions (*n* = 818), leaving a final analytic sample of 19,877 participants.

#### Statistical analysis

Descriptive statistics were used to summarise the prevalence of online food retail platform use by each country. Prevalence of online food retail platform use was defined as the weighted proportion of participants who reported using online food retail platforms within each country. Data were weighted with post-stratification sample weights, constructed using a raking algorithm with population estimates in each country, based on age group, sex at birth, region, ethnicity (except in Canada due to inconsistent collection methods used in national censuses) and education (except in Mexico due to difficulties recruiting low education participants) to improve the population representativeness of the results.

Although online food delivery use was assessed as an ordered categorical variable, it was dichotomised for our analysis to simplify interpretation and to avoid small sample sizes within categories.

Weighted pairwise comparisons of prevalence of use for different online food retail platforms were calculated using descriptive statistics. For each pair of online food retailers, the prevalence estimates were derived and their corresponding 95% confidence intervals were computed.

Logistic regression models were used to examine the association between sociodemographic characteristics (independent variables) and use of various types of online food retail platforms (dependent variables) for all countries combined. The dependent variables were binary indicators of whether participants reported using each platform type (yes/no).

Separate logistic regression models were used for each of the different online food retail platforms, and sample weights were applied to all analyses. To visually present the odds ratios and their corresponding 95% confidence intervals for each variable, forest plots were created using RStudio [22].

Additional logistic regression models were conducted to calculate odds ratios for the use of each platform stratified by country, with country included as a reference category (e.g., Australia, Canada, Mexico, UK, and the US), adjusted for age, sex, presence of children, BMI, ethnicity, cooking skills, frequency of food preparation, nutritional knowledge, and perceived income adequacy. Odds ratios and 95% confidence intervals were used to determine effect size and variance. Significance was determined at *p* < 0.05. All analyses were conducted using Stata SE 17 software (code available upon reasonable request) [23].

## Results

Overall, 51% of the sample was female, 32% of participants had children living at home aged under 18 years, and 27% of the sample belonged to the Millennial generation (aged 30–44) (Table 1). Mexico had the highest prevalence of children aged under 18 years living at home (50%), and the highest number of Millennial participants (31%). Canada had the lowest prevalence of children aged under 18 living at home (21%) and the UK had the lowest proportion of Millennial participants (25%).

**Table 1:** Weighted prevalence (%) of using online food retail platforms and sociodemographic characteristics, by country.

Variables		Country					
		Australia (*n* = 4003, 20.1%)	Canada (*n* = 4174, 21.0%)	Mexico (*n* = 3979, 20.0%)	United Kingdom (*n* = 3990, 20.1%)	United States (*n* = 3731, 18.8%)	Total (*n* = 19,877)
Prevalence of use of online ordering by food retail source (%, 95% CI)	Overall use of any retail or delivery platform	55.43 (53.73–57.13)	45.25 (43.41–47.15)	71.58 (69.85–73.25)	56.61 (54.93–58.29)	62.13 (60.31–63.92)	58.03 (57.23–58.82)
	Meals/food/drinks ordered online from restaurants (including through online food delivery platforms)	34.66 (33.00–36.36)	28.76 (27.12–30.48)	52.31 (50.49–54.13)	30.73 (29.20–32.33)	34.36 (32.65–36.14)	36.12 (35.35–36.89)
	Snacks/food/drinks ordered online from convenience stores (including through online food delivery platforms)	9.50 (8.47–10.64)	5.90 (5.01–6.95)	20.99 (19.62–22.45)	7.89 (7.01–8.89)	12.54 (11.39–13.83)	11.30 (10.80–11.82)
	Groceries ordered online from supermarkets with physical stores	28.26 (26.73–29.88)	18.72 (17.31–20.24)	28.18 (26.62–29.80)	31.90 (30.36–35.53)	33.10 (31.41–34.88)	27.90 (27.19–28.62)
	Groceries from online-only stores	9.20 (8.23–10.27)	6.90 (6.07–7.89)	15.08 (13.90–16.39)	10.73 (9.76–11.82)	16.00 (14.76–17.36)	11.49 (11.01–12.00)
	Meal kit delivery services	13.41 (12.29–14.65)	9.21 (8.07–10.44)	16.58 (15.31–17.96)	12.30 (11.22–13.51)	17.44 (16.07–18.90)	13.70 (13.1–14.3)
Sociodemographic characteristics (%)	Female	51.07 (49.34–52.79)	50.73 (48.85–52.61)	51.72 (49.93–53.54)	51.70 (50.01–53.41)	50.60 (48.76–52.46)	51.17 (50.37–52.00)
	Millennial generation	27.15 (25.59–28.79)	25.18 (23.60–26.84)	30.94 (29.36–32.58)	24.99 (23.54–26.52)	25.03 (23.43–26.71)	26.67 (25.96–27.39)
	Children under 18 years at home	30.68 (29.09–32.34)	21.28 (19.89–22.83)	50.03 (48.23–51.86)	28.90 (27.42–30.49)	28.28 (26.71–30.02)	31.80 (31.07–32.55)
	Adequate income	71.43 (69.85–72.97)	69.30 (67.44–71.10)	55.07 (53.26–56.88)	69.41 (67.78–70.98)	66.68 (64.90–68.42)	66.41 (65.63–67.18)
	Knowledgeable about nutrition	63.02 (61.33–64.67)	65.70 (63.83–67.53)	67.55 (65.83–69.21)	56.18 (54.48–57.86)	68.15 (66.37–69.88)	64.08 (63.30–64.85)
	Skilled at cooking	75.65 (74.13–77.12)	74.15 (72.41–75.81)	66.25 (64.52–67.93)	70.13 (68.55–71.66)	78.46 (76.87–79.97)	72.87 (72.15–73.59)
	Frequent food preparers	71.32 (69.69–72.90)	70.25 (68.43–72.00)	63.56 (61.82–65.27)	71.46 (69.89–72.98)	67.26 (65.46–69.00)	68.81 (68.05–69.56)
	Minority ethnic group	29.54 (27.75–31.40)	23.33 (21.86–24.87)	19.11 (17.61–20.71)	13.59 (12.38–14.90)	36.25 (34.35–38.20)	24.21 (23.46–24.96)
	BMI > 25	47.87 (46.15–49.59)	47.37 (45.50–49.25)	41.98 (40.18–43.80)	43.08 (41.40–44.77)	50.32 (48.47–52.17)	46.08 (45.28–46.89)

### Prevalence of online food retail platform use

Overall, 58% of participants reported using at least one online food retail platform (Table 1). Restaurants and supermarkets were the most common source of food that was ordered online or using an app (36% and 28% of participants reported ordering food online from restaurants and supermarkets in the past 30 days, respectively). Overall, 11% indicated they had ordered from an online-only grocery store in the previous 30 days. In total, 14% of participants had consumed food purchased from meal kit delivery services in the past 7 days. The pairwise comparisons of prevalence proportions revealed that the highest co-occurrence was between ‘online orders from restaurants’ and ‘groceries ordered online from physical supermarkets,’ with 15% of participants reporting using both services (95% CI: 14.37%–15.49%) (Supplementary Table 2).

Across the five countries, the prevalence of online ordering from restaurants (52%), and online convenience stores (21%) was highest in Mexico. The US had the highest prevalence of online orders from supermarkets (33%), online-only grocery stores (16%), and online meal kit delivery services (17%). Participants in Canada had the lowest prevalence of all online food retail platform uses (Table 1).

When comparing the participants’ use of online retail platforms across all countries (Supplementary Table 1), participants in Canada generally (but not always) had lower odds of using online retail platforms compared to all other countries. For example, participants in Canada had lower odds of ordering groceries online from physical supermarkets (OR: 0.62, 95% CI: 0.55–0.71) and ordering meal kits (OR: 0.66, 95% CI: 0.56–0.80) compared to participants in Australia. Participants in the US had greater odds of ordering online from most retailers, including online supermarkets when compared to Australia (OR: 1.31, 95% CI: 1.09–1.56).

### Associations between sociodemographic variables and online food retail platform use

#### Sociodemographic characteristics

**Fig. 1:**
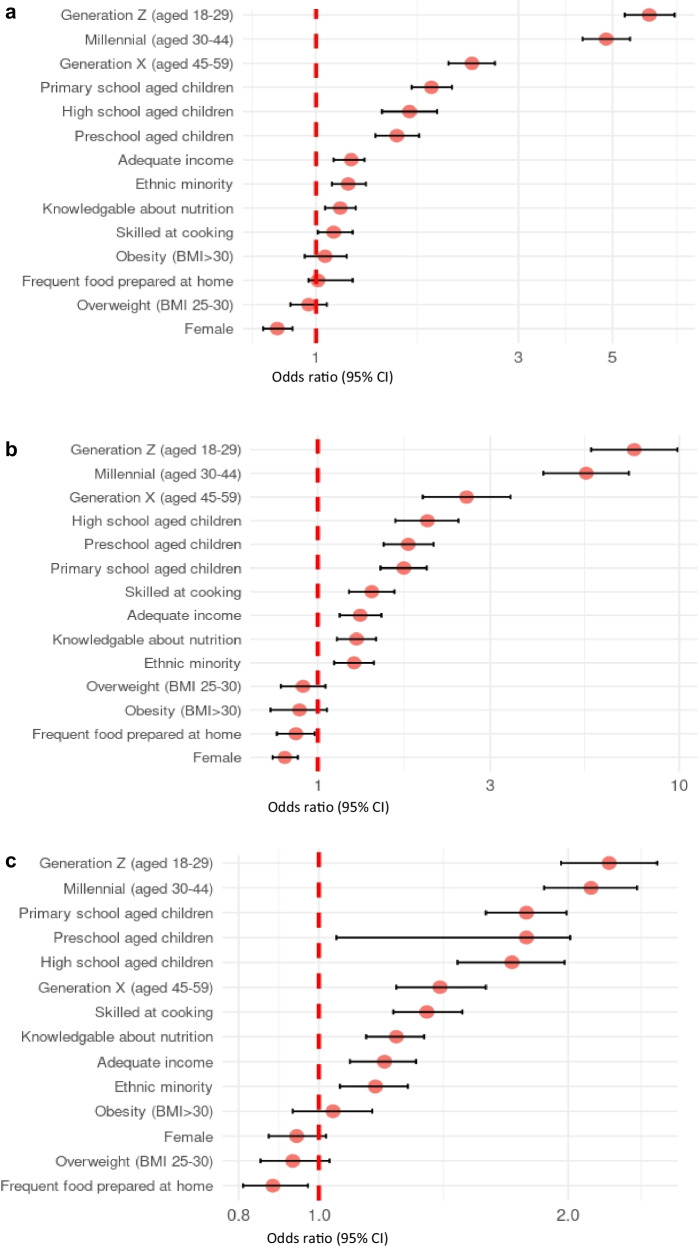

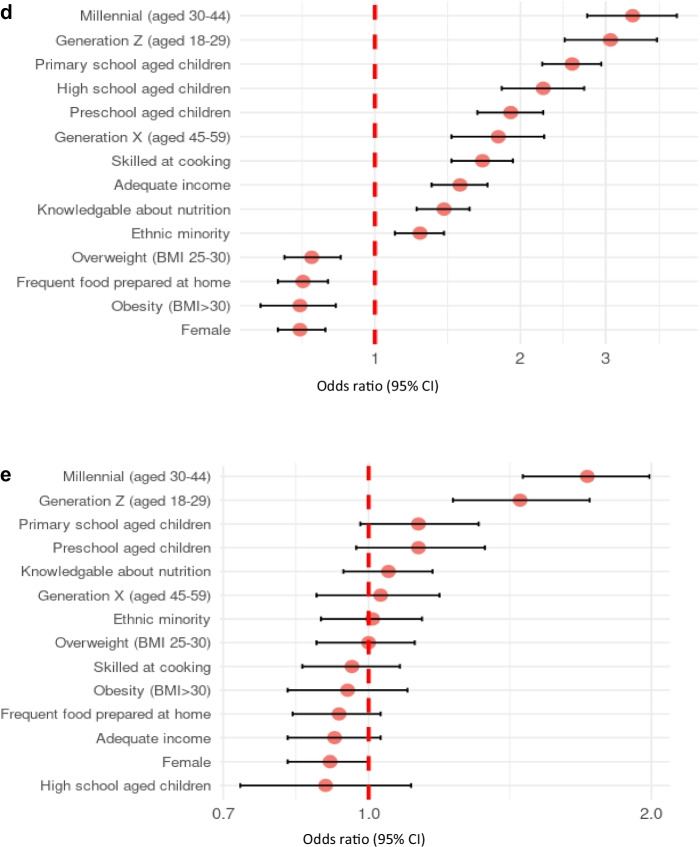
Associations between online food retail platform use and sociodemographic characteristics. **a** Online restaurants, **b** online convenience stores, **c** online supermarkets, **d** online-only grocery stores, **e** meal kits. Forest plots displaying adjusted odds ratios (ORs) with 95% confidence intervals from logistic regression analyses (*n* = 19,877). Reference categories (OR = 1): Age: Silent Generation and Baby Boomers (vs. Generation X, Millennials, Generation Z). Children at home: no children (vs. preschool-aged, primary school-aged, high school-aged children). Ethnicity: majority ethnicity (vs. minority ethnicity). BMI: underweight/normal weight (BMI 30). Sex: male (vs. female). Nutrition knowledge: not knowledgeable about nutrition (vs. knowledgeable about nutrition). Cooking skills: not skilled at cooking (vs. skilled at cooking). Food preparation frequency: infrequent food preparation at home (vs. frequent food preparation at home). Income adequacy: inadequate income (vs. adequate income).

Logistic regression models were used to examine the association between sociodemographic characteristics and the likelihood of using various types of online food retail platforms. The outcome variables were binary indicators of whether participants reported using each platform type (e.g., online orders from restaurants, convenience stores, online-only grocery stores, supermarkets, and meal kit delivery (Fig. 1a–e). Among the entire sample, the models revealed that females had significantly lower odds of making online orders from restaurants, convenience stores, and online-only grocery stores compared to males (Table 2). No significant difference in use of online meal kit delivery and online orders from supermarkets was observed between males and females.

**Table 2:** All countries analyses of associations between use of online food retail platforms, and sociodemographic characteristics.

Variable	Categories	Online restaurants OR (95% CI)	Online convenience stores OR (95% CI)	Online supermarkets OR (95% CI)	Online-only grocery stores OR (95% CI)	Meal kit delivery OR (95% CI)
Sex at birth	Male	*Reference* *n* = 3759	*Reference* *n* = 1252	*Reference* *n* = 2769	*Reference* *n* = 1405	*Reference* *n* = 1381
	Female	0.81 (0.75–0.88)* *n* = 3466	0.71 (0.63–0.79)* *n* = 977	0.94 (0.87–1.02) *n* = 2787	**0**.70 (0.63–0.79)* *n* = 1002	0.91 (0.82–1.00) *n* = 1280
BMI (kg/m^2^)	Underweight/ normal weight (BMI < 25 kg/m^2^)	*Reference* *n* = 2919	*Reference* *n* = 954	*Reference* *n* = 2233	*Reference* *n* = 1111	*Reference* *n* = 1050
	Overweight (BMI 25–29.9 kg/m^2^)	0.96 (0.87–1.06) *n* = 1808	0.91 (0.79–1.05) *n* = 517	0.93 (0.85–1.03) *n* = 1330	0.74 (0.65–0.84)* *n* = 520	1.00 (0.88–1.12) *n* = 706
	Obesity (BMI > 30 kg/m^2^)	1.05 (0.94–1.18) *n* = 1193	0.89 (0.74–1.06) *n* = 288	1.04 (0.93–1.16) *n* = 949	0.70 (0.58–0.83)* *n* = 275	0.95 (0.82–1.10) *n* = 450
	Missing value	1.04 (0.93–1.17) *n* = 1305	0.98 (0.84–1.14) *n* = 470	1.32 (1.05–1.78)* *n* = 1044	1.07 (0.92–1.23) *n* = 501	1.02 (0.88–1.19) *n* = 455
Children at home	No children	*Reference* *n* = 3720	*Reference* *n* = 897	*Reference* *n* = 2882	*Reference* *n* = 920	*Reference* *n* = 1618
	Preschool aged	1.55 (1.38–1.75)* *n* = 1229	1.78 (1.52–2.09)* *n* = 491	1.78 (1.57–2.01)* *n* = 958	1.91 (1.63–2.23)* *n* = 470	1.13 (0.97–1.33) *n* = 369
	Primary school aged	1.87 (1.68–2.09)* *n* = 1649	1.73 (1.49–2.00)* *n* = 619	1.78 (1.59–1.99)* *n* = 1239	2.56 (2.22–2.94)* *n* = 791	1.13 (0.98–1.31) *n* = 505
	High school aged	1.66 (1.43–1.93)* *n* = 627	2.01 (1.64–2.45)* *n* = 222	1.71 (1.47–1.98)* *n* = 477	2.23 (1.83–2.71) *p* = 0 *n* = 26	0.90 (0.73–1.11) *p* = 0.33 *n* = 169
Generation	Silent and Baby Boomer (aged 60+)	*Reference* *n* = 695	*Reference* *n* = 90	*Reference* *n* = 800	*Reference* *n* = 171	*Reference* *n* = 575
	Generation X (aged 45–59)	2.33 (2.05–2.64)* *n* = 1635	2.58 (1.95–3.41)* *n* = 381	1.40 (1.24–1.59)* *n* = 1294	1.80 (1.44–2.24)* *n* = 458	1.03 (0.88–1.19) *n* = 595
	Millennial (aged 30–44)	4.83 (4.25–5.50)* *n* = 2692	5.53 (4.21–7.26)* *n* = 940	2.13 (1.87–2.42)* *n* = 2009	3.41 (2.75–4.21)* *n* = 1118	1.71 (1.46–1.99)* *n* = 885
	Generation Z (aged 18–29)	6.10 (5.34–7.00)* *n* = 2204	7.51 (5.71–9.88)* *n* = 818	2.24 (1.96–2.56)* *n* = 1444	3.07 (2.47–3.83)* *n* = 660	1.45 (1.23–1.72)* *n* = 606
Food preparation at home^a^	Not frequent	*Reference* *n* = 2468	*Reference* *n* = 842	*Reference* *n* = 1831	*Reference* *n* = 936	*Reference* *n* = 921
	Frequent	1.01 (0.96–1.22) *n* = 4757	0.87 (0.77–0.98)* *n* = 1387	0.88 (0.81–0.97)* *n* = 3725	0.71 (0.63–0.80)* *n* = 1471	0.93 (0.83–1.03) *n* = 1740
Cooking skills^b^	Unskilled	*Reference* *n* = 1763	*Reference* *n* = 462	*Reference* *n* = 1220	*Reference* *n* = 404	*Reference* *n* = 732
	Skilled	1.10 (1.01–1.22)* *n* = 5462	1.41 (1.22–1.63)* *n* = 1767	1.35 (1.23–1.49)* *n* = 4336	1.67 (1.44–1.93)* *n* = 2003	0.96 (0.85–1.08) *n* = 1929
Nutrition knowledge^c^	Not knowledgeable	*Reference* *n* = 1763	*Reference* *n* = 462	*Reference* *n* = 1220	*Reference* *n* = 404	*Reference* *n* = 896
	Knowledgeable	1.14 (1.05–1.24)* *n* = 5462	1.28 (1.13–1.45)* *n* = 1767	1.24 (1.14–1.34)* *n* = 4336	1.39 (1.22–1.57)* *n* = 2003	1.05 (0.94–1.17) *n* = 1765
Perceived income	Inadequate	*Reference* *n* = 1763	*Reference* *n* = 462	*Reference* *n* = 1220	*Reference* *n* = 404	*Reference* *n* = 890
	Adequate	1.21 (1.10–1.32)* *n* = 5462	1.31 (1.15–1.50)* *n* = 1767	1.20 (1.09–1.31)* *n* = 4336	1.50 (1.31–1.71)* *n* = 2003	0.92 (0.82–1.03) *n* = 1771
Ethnicity	Majority	*Reference* *n* = 5473	*Reference* *n* = 1621	*Reference* *n* = 4231	*Reference* *n* = 1720	*Reference* *n* = 2107
	Minority	1.19 (1.09–1.31)* *n* = 1752	1.26 (1.11–1.43)* *n* = 608	1.17 (1.06–1.28)* *n* = 1325	1.24 (1.10–1.39)* *n* = 687	1.01 (0.89–1.14) *n* = 554

* significant *p* < 0.05.

^a^“Frequent” defined as preparing food least 3–4 times per week.

^b^“Skilled” defined as self-reported cooking skills being excellent, very good, or good.

^c^“Knowledgeable” defined as self-reported nutrition knowledge being extremely, very, or somewhat knowledgeable.

Millennial and Generation Z age groups had significantly greater odds of using all online food retail platforms compared to the Baby Boomer/Silent Generation participants. As an example, for online restaurants, Generation Z had the greatest odds of use (OR: 6.10, 95% CI: 5.34–7.00), followed by Millennials (OR: 4.83 95% CI 4.25–5.50) when compared to the Baby Boomer/Silent Generation participants (Fig. 1a–e).

Participants with primary school aged children had higher odds of ordering groceries online from supermarkets (OR: 2.56, 95% CI: 2.22–2.94) and online-only grocery stores (OR: 2.56, 95% CI 2.22–2.94) compared to those without children. Participants with adequate income had greater odds of making online orders from restaurants (OR: 1.21, 95% CI: 1.10–1.32), convenience stores (OR: 1.31, 95% CI: 1.15–1.50), supermarkets (OR: 1.20, 95% CI: 1.09–1.31), and online-only grocery stores (OR: 1.50, 95% CI: 1.31–1.71) than those who reported inadequate income.

Participants who identified as belonging to an ethnic minority group had greater odds of making online orders from restaurants (OR: 1.19, 95% CI: 1.09–1.31), convenience stores (OR: 1.26, 95% CI: 1.11–1.43), supermarkets (OR: 1.17, 95% CI: 1.06–1.28), and online-only grocery stores (OR: 1.24, 95% CI: 1.10–1.39) than those identifying as an ethnic majority (Table 2). Participants with self-reported BMI of 25–30 kg/m^2^ and greater than 30 kg/m^2^ had lower odds of using online-only grocery stores (OR: 0.74, 95% CI: 0.65–0.84, and OR: 0.70, 95% CI 0.58–0.83, respectively), than those with a BMI less than 25 kg/m^2^.

#### Behavioural characteristics

Participants classified as frequently preparing meals at home had lower odds of making online orders from convenience stores (OR: 0.87, 95% CI: 0.77–0.98), supermarkets (OR: 0.88, 95% CI: 0.81–0.97), and online-only grocery stores (OR: 0.71, 95% CI: 0.63–0.80) compared to those who did not prepare meals at home frequently. Participants who reported they were skilled at cooking had greater odds of ordering food online from restaurants (OR: 1.10, 95% CI: 1.01–1.22), convenience stores (OR: 1.41, 95% CI: 1.22–1.63), supermarkets, (OR: 1.35, 95% CI: 1.23–1.49), and online-only grocery stores (OR: 1.67, 95% CI: 1.44–1.93) than participants who reported

Participants classified as knowledgeable about nutrition had greater odds of making online orders from restaurants (OR: 1.14, 95% CI: 1.05–1.24), convenience stores (OR: 1.28, 95% CI: 1.13–1.45), supermarkets (OR: 1.24, 95% CI: 1.14–1.34), and online-only grocery stores (OR: 1.39, 95% CI: 1.22–1.57) than those classified as not knowledgeable.

## Discussion

To our knowledge, this is the first paper to describe the use of multiple different online food retail platforms across multiple countries. We found that OFD platforms were used most frequently for online orders from restaurants (36%), followed by online grocery orders from supermarkets with physical stores (28%). By country, Mexico had the highest prevalence of online ordering from restaurants (52%) and convenience stores (21%). The US had the highest prevalence of online ordering from supermarkets (33%), online-only grocery stores (16%), and meal kit delivery services (18%). Significant associations were found between use of online food retail platforms and being male, younger age, and having children present in the home. Adequate income was generally associated with greater odds of using all online food retail. Those who frequently prepared meals at home were significantly less likely to use online convenience stores, online supermarkets, and online-only grocery stores.

### Comparison with previous research

Our results are similar to other analyses focusing on OFD platforms, based on 2018 IFPS data, which found that men (OR: 1.50, 95% CI: 1.35–1.66), those who identify as an ethnic minority (OR: 1.57, 95% CI: 1.38–1.78), and those who live with children aged under 18 years (OR: 2.71, 95% CI: 2.44–3.01) had higher odds of using OFDPs for restaurants and takeaways [24]. Similarly, a study examining adults aged 18–25 in the United States found that more frequent use of OFD platforms was associated with identifying as an ethnic minority, experiencing food insecurity, and having higher perceived social status [25]. Similar to our findings, a UK study examining online grocery shopping found that more affluent households, and those aged 25–44 were the most likely to shop online for groceries to be delivered [26]. However, a previous study of over 34,000 Italian respondents found a positive relationship between purchasing groceries online and being well-educated, female, and experiencing obesity, contrary to our results which found no association between being female or BMI above 30 kg/m^2^ and ordering online from supermarkets [27].

### Generational trends

Our study found a significant relationship between ordering from online food retail platforms and being a Millennial (30–44 years old) or member of Generation Z (18–29 years old). Both of these generations are characterised by their willingness to try new technology, considering themselves “early adopters” [28, 29]. Additionally, these age groups typically find convenience worth paying more for, compared with older generations [28]. Some evidence suggests that Millennials parents are more time-poor than previous generations [30], due in part to the higher likelihood of both adults in a typical household working outside the home. This could mean that options that save time (such as ordering groceries online) are more appealing to them than older generations who are no longer raising young children. Some have suggested that online food retail platforms may also allow for greater opportunities to try new foods, rather than being confined to the products available at retail outlets within a neighbourhood food environment [4]. Millennials [31] and Generation Z [32] have been described as more adventurous than previous generations in terms of trying new foods, which could also make online ordering attractive.

### Country-specific trends and health implications

Prevalence of use of all online food retail was highest in Mexico, reflecting a general trend of increased e-commerce sales in the country. In 2023, Mexico experienced a 25% growth in e-commerce sales (including online food retail), compared to a global average of 10% [33]. Additionally, the number of internet users in Mexico either shopping online, or shopping and making payments online, rose from 6.08 million in 2015 to 28.46 million people in 2022 [34]. However, our study does not clearly explain the reasons behind our observed high prevalence of online food retail use, so further research examining this in more detail is warranted.

Prevalence of online ordering from supermarkets, online-only grocery stores, and meal kit delivery services were highest in the US. This may be explained by the food environment characteristics in the US, which have been described as often lacking in retailers that sell fresh foods, especially in neighbourhoods that are predominantly non-white or lower socioeconomic position [35]. Associations have been found between characteristics of the US food environment (such as access to fast food restaurants and convenience stores, or lack of access to supermarkets) and obesity [36]. Moreover, the online grocery landscape in the US is dominated by large corporations, such as Walmart and Amazon [37]. These retailers have vast logistic networks and marketing strategies to facilitate deliveries across wide geographic areas [38], potentially increasing access to groceries for those in areas that are not well served by physical stores. Although operating globally, meal kit brand Hello Fresh reported 66% of its 2023 revenue from North America [39]. Meal kits can fulfil a similar niche as online grocery in that they are able to provide fresh ingredients as an alternative to takeaway foods [6]. Interestingly, in our study ordering from meal kit delivery services was not found to be associated with participant income, despite their increased cost relative to normal groceries [40]. This could be due to the marketing strategies of meal kit companies (advertising benefits including reduced food waste and stress surrounding meal planning) which may appeal to a broad range of customers, regardless of income. Further research is needed to better understand the factors that drive meal kit usage across different income groups, particularly to understand how marketing may influence consumer perceptions.

Prevalence of use of online food retail platforms was lowest in Canada. The costs associated with using online food retail platforms (including delivery and service fees) have been identified as a barrier to Canadians using these services [41]. Additionally, the distance required to travel to physical food retailers in Canada [42] may mean different food purchasing habits have been adopted by the local population, perhaps utilising larger food shopping events less often rather than more frequent, small purchases which would align more closely with the purchasing model commonly used by online food retail platforms.

### Other sociodemographic characteristics

Results from our study suggest that users of online food retail platforms were more likely to perceive that they have good cooking skills, and are knowledgeable about nutrition, but they typically prepared food at home less than two times per week. This may be related to potential implications of online food retail platforms on diet and health as home cooked meals are beneficial to health, shown in a UK study examining adults (*n* = 11,396) who ate home cooked meals at least five times per week and found that they had a greater consumption of fruits and vegetables, and were more likely to have a normal range BMI (less than 25 kg/m^2^) and body fat percentage, compared to those who prepared meals three times per week or less [43]. A cross sectional study of US adults (*n* = 12,842) found that those who ate meals that were entirely home cooked had a 26% lower odds of obesity than those who ate some or no home cooked meals (OR 0.74 95% CI = 0.62–0.88) [44]. However, the determinants of preparing food at home have been theorised as being more complex than simply having the required cooking skills [45]. Adults and families are more time-poor now than previously, which reduces available time for food preparation and may see them relying on prepared foods [46], such as those available through online food retail platforms. Generally, females are responsible for food purchasing and preparation [45], particularly in families [47]. Our findings show that males are more likely than females to use online food retail platforms to order from restaurants, convenience stores, and online-only grocery stores.

### Policy and research implications

Given that diet is a leading risk factor for chronic disease [48], and the relatively high prevalence of online food retail platform use, efforts to improve population diets need to ensure that online food retail platforms support good health and nutrition. For example, providing nutritional information in online settings that are equivalent to physical food retail stores can allow customers to make an informed choice about their purchases [49]. Ensuring that platforms are not promoting unhealthy options through algorithmic boosting, or using pricing strategies can make healthy options more visible and appealing may also be useful health promoting strategies. More detailed research understanding how families are using online food retail platforms to meet their needs could be explored in future research, as it is important to ensure that unhealthy food purchasing behaviours are not being normalised through use of these platforms.

Policies that aim to improve the healthiness of online food retail platforms may also benefit people belonging to ethnic minority groups, as we found they have a higher-odds of making online orders from restaurants, convenience stores, supermarkets, and online-only grocery stores, compared to ethnic majority groups. Ethnic minority groups are often priority populations for public health, as they can experience poorer nutrition outcomes, have a greater prevalence of obesity, and are more likely to live in neighbourhoods with lower availability of healthy food [50, 51]. Given that online food retail platforms have been shown to promote unhealthy food options [13], and reduce the desire to purchase fresh items like fruit due to quality concerns [16], their potential role in perpetuating or exacerbating dietary inequalities is of concern. Identifying factors that influence people from ethnic minority groups to use online food retail platforms, and their perceptions as to how to improve the healthiness of offerings available could be studied in further research.

### Strengths and limitations

This study has several strengths, including the use of data from multiple countries which allows for comparisons between population groups to determine if a unique social or political circumstance has caused greater demand for online food retail platforms in one country. Additionally, the sample size is large, and our analysis used weighted samples to ensure the results more closely represent those of each country’s general population.

However, this study is also subject to limitations. All measures were self-reported, and some (such as cooking skills and nutrition knowledge) relied on participants’ perceptions. These data are not objectively measured and may be influenced by social desirability bias, though this may be reduced somewhat by the online nature of the IFPS. This study focused on the frequency of online food purchasing and did not account for the size or nature of purchases (e.g., monetary value, number of items, or specific types of food), which may limit the depth of understanding of online food purchasing. Additionally, we did not include survey items on participants’ use of, or exposure to, nutrition information within online food retail platforms, limiting its ability to explore potential implications for dietary intake and health outcomes or to assess how these platforms may influence consumer behaviour. This study relied on a complete-case analysis, which assumes that missing data is missing at random. However, as we did not conduct formal tests to verify this assumption, the potential for bias due to non-random missingness cannot be ruled out. In addition, our study is limited by the differing timescales used for measuring meal kit use (7 days) and other online food retail services (30 days), which reflects the structure of the IFPS survey and may limit direct comparability between services. This discrepancy could be addressed in future studies. Finally, the survey is conducted online so the sample population is likely familiar with digital technology and may represent a group that is more technologically savvy, which may affect their likelihood of previously using online ordering to purchase food. Future studies using non-online data collection methods would be of interest as a comparator.

## Conclusion

This study quantified the substantial prevalence of online ordering from restaurants and supermarkets across five middle- and high-income countries. Across all countries, 58% of participants used at least one type of online food retail platform, with notable differences in usage rates by platform type. The analysis revealed significant associations between online food retail use and various sociodemographic and behavioural factors, such as sex at birth, age, presence of children in the home, self-reported cooking skills and nutritional knowledge, frequency of food prepared at home, and identifying as being from an ethnic minority. Efforts to improve population diets need to ensure that online food retail platforms support good health and nutrition.

## Supplementary information

Supplementary Table 1, Supplementary Table 2

## Data Availability

The datasets analysed during the current study are available from the corresponding author on reasonable request.
