# Supplementary material for: Prevalence of online food delivery platforms, meal kit delivery, and online grocery use in five countries: an analysis of survey data from the 2022 International Food Policy Study
Source: Int J Obes (Lond). 2025 Apr 13;49(7):1307–16. doi: 10.1038/s41366-025-01771-z (PMC12283340; doi:10.1038/s41366-025-01771-z)
Supplement: Supplementary file 1 — Supplementary Table 1, Supplementary Table 2 [file 41366_2025_1771_MOESM1_ESM.docx]

## Supplementary Files:

| Variable | Categories | Online orders from restaurants,  OR (95% CI) | Online orders from convenience stores  OR (95% CI) | Groceries ordered online from physical supermarkets  OR (95% CI) | Groceries ordered from online only stores,  OR (95% CI) | Meal kit delivery  OR (95% CI) |
| --- | --- | --- | --- | --- | --- | --- |
| Country | Australia | *Reference*  *n=1296* | *Reference*  *n=345* | *Reference*  *n=1087* | *Reference*  *n=360* | *Reference*  *n=534* |
|  | Canada | **0.82 (0.73 – 0.92) ***  **n=1188** | **0.62 (0.49 – 0.77) ***  **n=212** | **0.62 (0.55 – 0.71) ***  **n=757** | 0.87 (0.72 – 1.06)  n=295 | **0.66 (0.56-0.80) ***  **n=337** |
|  | Mexico | **1.70 (1.51 – 1.90) ***  **n=2172** | **2.13 (1.81 – 2.50) ***  **n=881** | **0.80 (0.71 – 0.90) ***  **n=1197** | **1.54 (1.31 – 1.82) ***  **n=657** | **1.18 (1.02-1.38) ***  **n=666** |
|  | UK | 0.91 (0.81 – 1.01)  n=1256 | 0.89 (0.74 – 1.08)  n=315 | **1.30 (1.16 – 1.47) ***  **n=1277** | **1.43 (1.20 – 1.69) ***  **n=463** | 0.93 (0.80-1.08)  n=473 |
|  | USA | 0.99 (0.88 – 1.12)  n=1313 | **1.31 (1.09 – 1.56) ***  **n=476** | **1.25 (1.11 – 1.41) ***  **n=1238** | **2.00 (1.69 – 2.36) ***  **n=632** | **1.40 (1.21-1.61) ***  **n=651** |
|  | Canada | *Reference* | *Reference* | *Reference* | *Reference* | *Reference* |
|  | Mexico | **2.07 (1.83 – 2.34) *** | **3.45 (2.81 – 4.25) *** | **1.28 (1.12 – 1.47) *** | **1.77 (1.47 – 2.13) *** | **1.78 (1.48 – 2.13) *** |
|  | UK | 1.10 (0.98 – 1.25) | **1.45 (1.15 – 1.82) *** | **2.10 (1.84 – 2.39) *** | **1.64 (1.35 – 1.98) *** | **1.40 (1.17 – 1.68) *** |
|  | USA | **1.21 (1.07 – 1.37) (** | **2.12 (1.71 – 2.63) *** | **2.01 (1.77 – 2.30) *** | **2.29 (1.91 – 2.75) *** | **2.10 (1.75 – 2.49) *** |
|  | Mexico | *Reference* | *Reference* | *Reference* | *Reference* | *Reference* |
|  | UK | **0.53 (0.48 – 0.60) *** | **0.42 (0.35 – 0.50) *** | **1.63 (1.45 – 1.84) *** | 0.92 (0.79 – 1.09) | **0.79 (0.68 – 0.92) *** |
|  | USA | **0.59 (0.52 – 0.66) *** | **0.61 (0.52 – 0.72) *** | **1.57 (1.39 – 1.78) *** | **1.30 (1.11 – 1.51) *** | **1.18 (1.01 – 1.37) *** |
|  | UK | *Reference* | *Reference* | *Reference* | *Reference* | *Reference* |
|  | USA | 1.10 (0.97 – 1.24) | **1.47 (1.22 – 1.77) *** | 0.96 (0.85 – 1.08) | **1.40 (1.19 – 1.64) *** | **1.50 (1.29 – 1.74) *** |

*Supplementary Table 1: Associations between country and prevalence of online food retail platform use*

**significant at <0.05*

|  | Online orders from restaurants | Online orders from convenience stores | Groceries ordered online from physical supermarkets | Groceries ordered from online only stores, | Meal kit delivery |
| --- | --- | --- | --- | --- | --- |
| Online orders from restaurants | NA | 8.21 (7.77 – 8.64) | 14.93 (14.37 – 15.49) | 7.41 (7.02 – 7.82) | 5.52 (5.17 – 5.89) |
| Online orders from convenience stores | 8.21 (7.77 – 8.64) | NA | 7.03 (6.62 – 7.44) | 3.92 (3.63 – 4.23) | 2.05 (1.82 – 2.28) |
| Groceries ordered online from physical supermarkets | 14.93 (14.37 – 15.49) | 7.03 (6.62 – 7.44) | NA | 6.84 (6.44 – 7.23) | 4.32 (3.99 – 4.66) |
| Groceries ordered from online only stores, | 7.41 (7.02 – 7.82) | 3.92 (3.63 – 4.23) | 6.84 | NA | 2.10 (18.80 – 2.32) |
| Meal kit delivery | 5.52 (5.17 – 5.89) | 2.05 (1.82 – 2.28) | 4.32 (3.99 – 4.66) | 2.10 (18.80 – 2.32) | NA |

###### Supplementary Table 2: Pairwise comparisons of weighted prevalence (%, with 95% confidence intervals) for different online food retail platforms.
